# 5-Cyclo­propyl-1-[(4-methyl­benzene)­sulfon­yl]indole-2-carbaldehyde

**DOI:** 10.1107/S2414314626009272

**Published:** 2026-09-15

**Authors:** Veeranjaneyulu Avula, Keerthana V.K., N.D. Yash, Umamaheshwar S., Nagendra Govindappa, Jayaraman Muthukumaran, Kuppuswamy Arumugam, Surya Prakash Rao H.

**Affiliations:** a Department of Chemistry REVA University, Rukmini Knowledge Park Kattigenahalli Yelahanka Bangalore 560064 Karnataka India; b Teadus Pharma, Plot No. 3B, Type-1, Sy. No.386, TSIIC Industrial Estate, Prashanth Nagar, Kukatpally, Hyderabad 500072, Telangana, India; c Department of Biotechnology, Sharda School of Bio-Science & Technology Sharda University Greater Noida India; d Centre for Bio-Organic Chemistry REVA Research Centre REVA University, Rukmini Knowledge Park Kattigenahalli Yelahanka Bangalore 560064 Karnataka India; e Department of Chemistry, Wright State University, 3640 Colonel Glenn Hwy., Dayton, OH 45435, USA; University of Antofagasta, Chile

**Keywords:** single-crystal X-ray diffraction, indole derivative, crystal structure, Hirshfeld surface analysis, π–π stacking inter­actions, C—H⋯O hydrogen bonding

## Abstract

The crystal structure of the title compound, C_19_H_17_NO_3_S, was determined by single-crystal X-ray diffraction. The compound crystallizes in the monoclinic crystal system with space group *P*2_1_/*c*. The indole ring is essentially planar, with cyclo­propyl, aldehyde and *p*-toluene­sulfonyl groups attached at the C5, C2 and N1 positions, respectively. The aldehyde substituent is nearly coplanar with the indole ring, while the cyclo­propyl and *p*-tolyl rings are almost perpendicular to it. The crystal packing is mainly influenced by slipped π–π stacking inter­actions, along with weak C—H⋯O inter­actions involving the sulfonyl oxygen atoms. Hirshfeld surface analysis shows that H⋯H (46.6%), O⋯H/H⋯O (23.5%), C⋯H/H⋯C (16.0%) and C⋯C (8.0%) contacts make the major contributions to the crystal packing.

## Abstract 2

The crystal structure of 5-cyclo­propyl-1-tosyl-1*H*-indole-2-carbaldehyde shows a nearly planar indole core, with slipped π–π stacking inter­actions contributing to the crystal packing. Hirshfeld surface analysis indicates that H⋯H, O⋯H/H⋯O and C⋯H/H⋯C contacts make the major contributions to the crystal packing.

## Graphical abstract

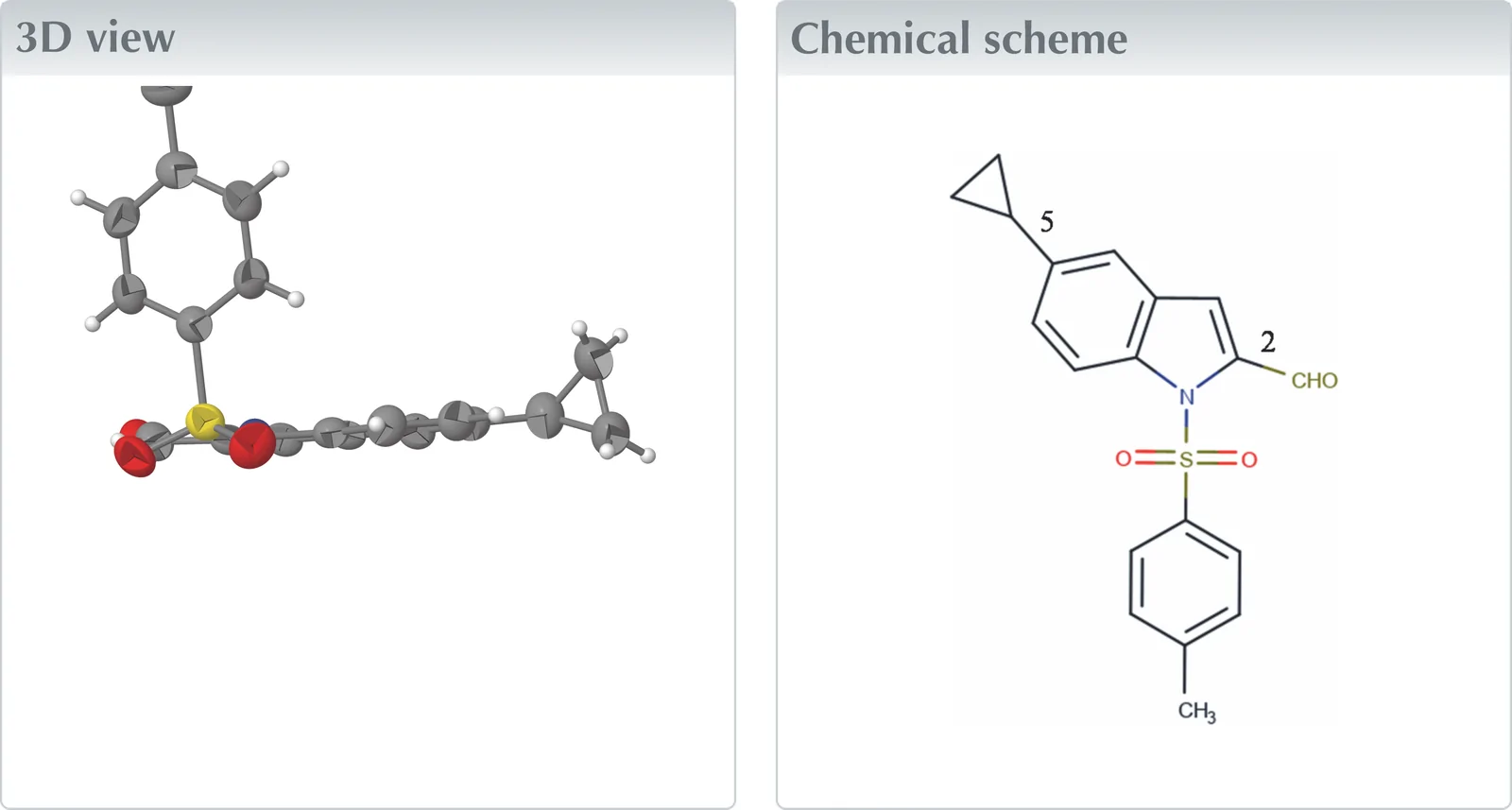

## Structure description

**Figure 1:**
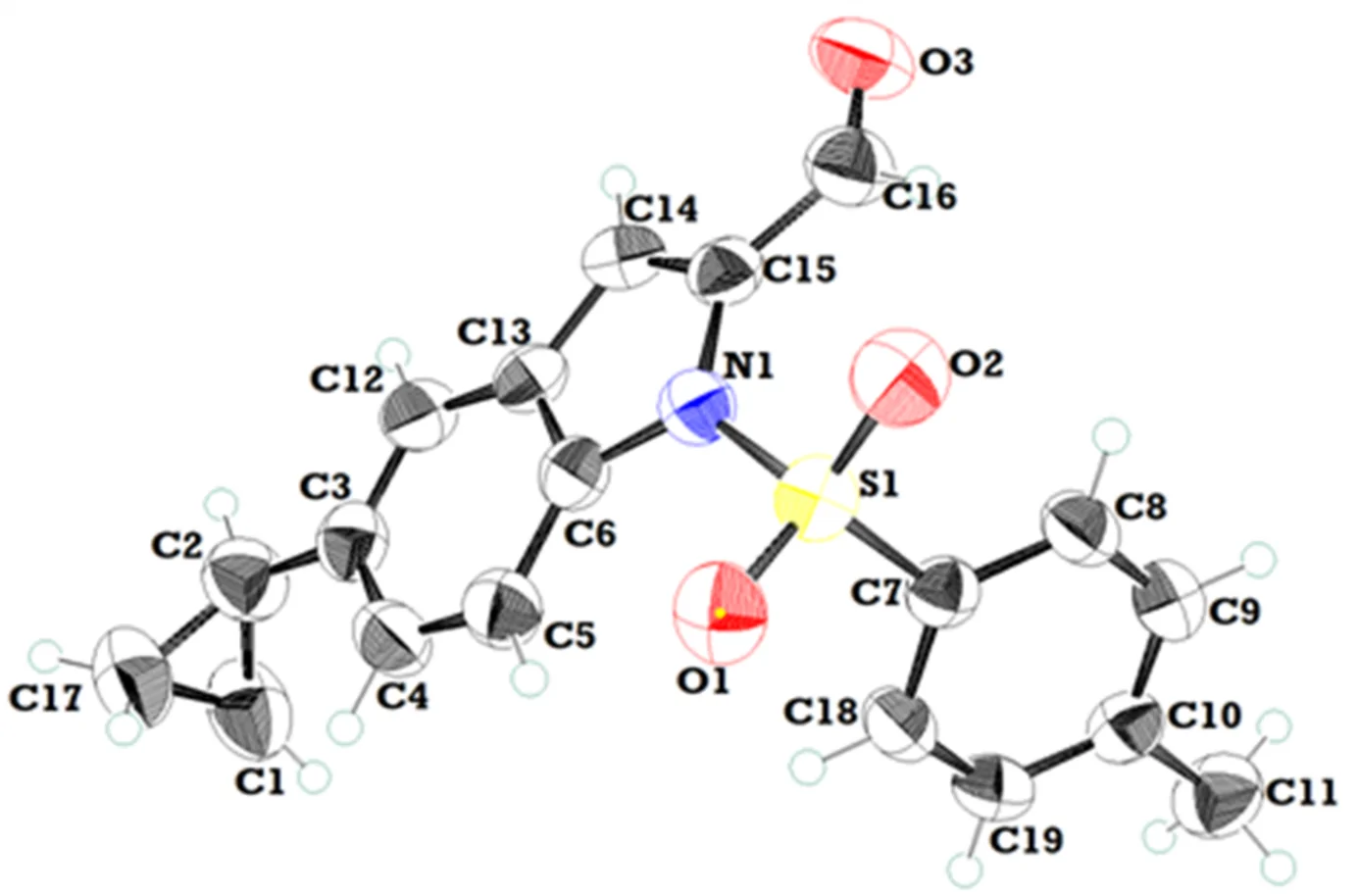
Mol­ecular structure of the title compound showing the atom-numbering scheme with displacement ellipsoids drawn at the 50% probability level

Indole derivatives continue to attract considerable attention because of their diverse biological activities and their widespread occurrence in natural products and therapeutic agents (Sundberg, 2012 ▸; Gribble, 2016 ▸; Kumar *et al.*, 2022 ▸). Owing to their structural versatility, indole-based compounds have become privileged scaffolds in medicinal chemistry, and structural modification of the groups attached to the indole nucleus can influence mol­ecular conformation, inter­molecular inter­actions and physicochemical properties (Sravanthi & Manju, 2016 ▸; Zeng *et al.*, 2024 ▸; Yang *et al.*, 2026 ▸; Drăgoi *et al.*, 2026 ▸). Among indole-incorporated small organic mol­ecules (SOMs), those containing a cyclo­propane ring have attracted attention for tuning the ADMET properties of drug candidates (Foote *et al.*, 2013 ▸; Tran & Henary, 2022 ▸; Hassam *et al.*, 2012 ▸). In view of the inter­est in cyclo­propyl- and indole-containing SOMs, we determined the solid-state structure of the title compound (Fig. 1 ▸) and describe its structural features here.

**Figure 2:**
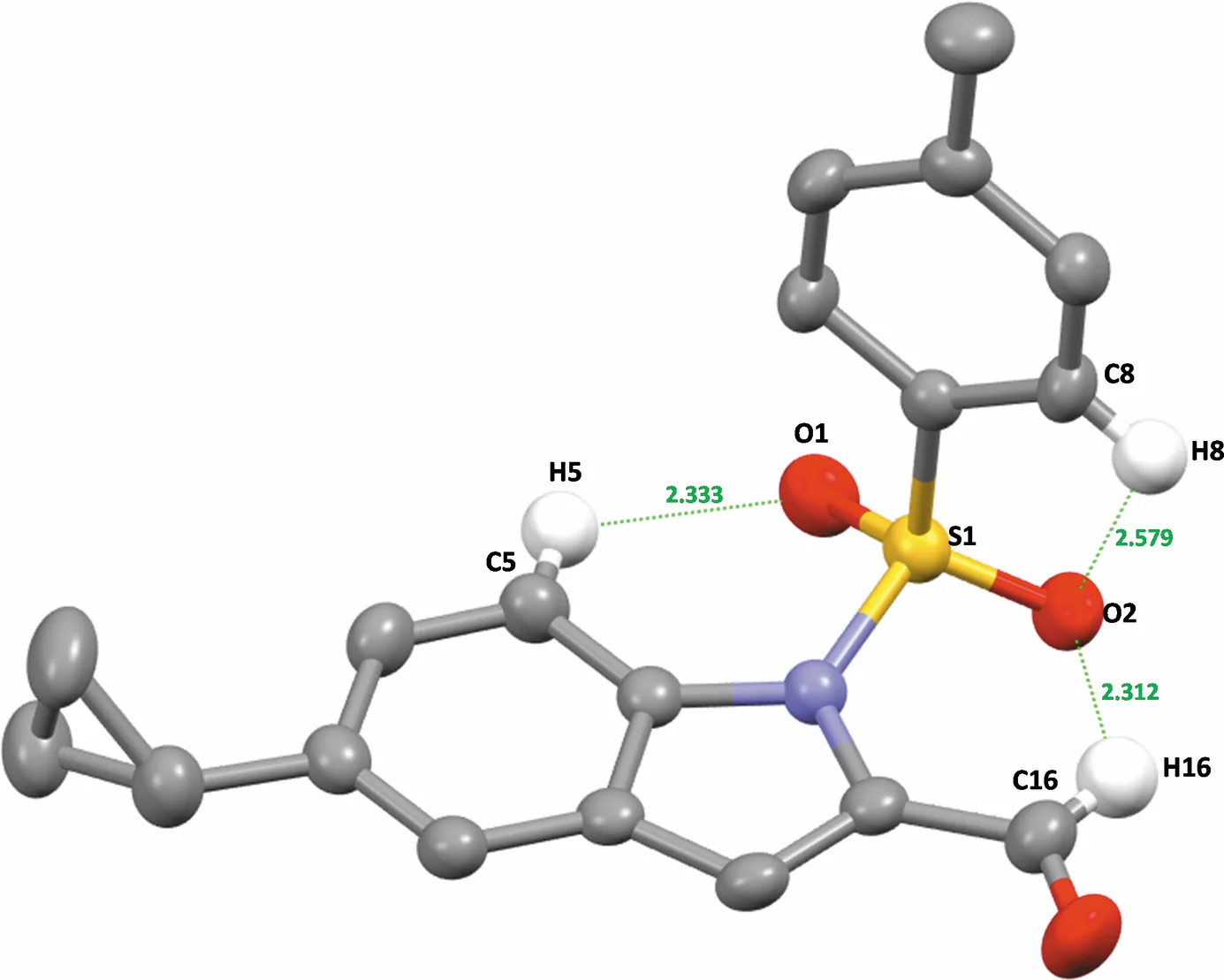
Mol­ecular structure of title compound showing the intra­molecular weak C—H⋯O inter­actions involving the sulfonyl oxygen atoms, which contribute to conformational stabilization. The atomic labelling scheme is shown for clarity.

The mol­ecular structure of the title compound is shown in Fig. 2 ▸. The compound crystallizes in the monoclinic space group *P*2_1_/*c*, with one mol­ecule in the asymmetric unit. The mol­ecule consists of an indole ring substituted by a cyclo­propyl group at the C5 position, an aldehyde group at C2, and a *p*-toluene­sulfonyl (tos­yl) group attached to atom N1. The indole ring system is essentially planar with an r.m.s. deviation of 0.019 Å, whereas the overall mol­ecular conformation is non-coplanar because of the orientation of the substituent groups. The sulfur atom adopts the expected distorted tetra­hedral geometry, being coordinated by two oxygen atoms, one nitro­gen atom and one carbon atom. The S1—O1 [1.421 (2) Å], S1—O2 [1.422 (2) Å] and S1—N1 [1.684 (3) Å] bond lengths are typical of aromatic sulfonamide derivatives. Likewise, the widened O1—S1—O2 angle of 120.73 (15)° is characteristic of sulfonyl-containing compounds and is consistent with the geometry commonly observed for aromatic sulfonamides.

**Figure 3:**
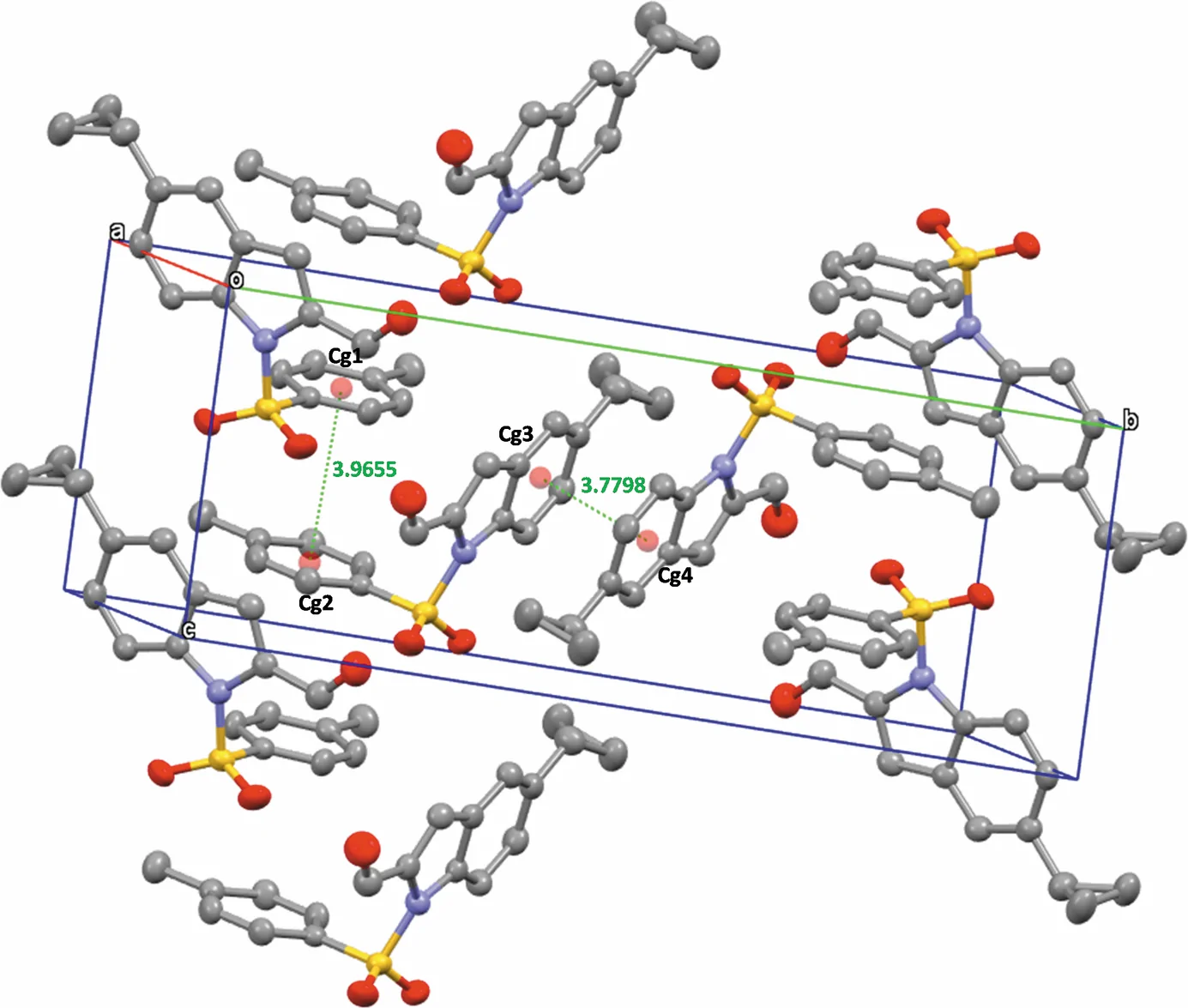
Crystal packing of the title compound showing significant slipped π–π stacking inter­actions between neighboring aromatic rings contributing to supra­molecular assembly and mol­ecular packing [centroid–centroid distances *Cg*1⋯*Cg*2 = 3.9655 (18) and *Cg*3⋯*Cg*4 = 3.7798 (18) Å where *Cg*1 and *Cg*2 are the centroids of the C7–C10/C18/C19 ring and its syemmetry-generated counterpart at (*x*, 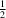 − *y*, −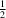 + *z*) and *Cg*2 and *Cg*3 are the centroids of the C3–C6/C12/C13 ring and its symmetry-generated counterpart at (1 − *x*, −*y*, −*z*)]. Hydrogen atoms are omitted for clarity.

Least-squares plane analysis shows that the aldehyde group is nearly coplanar with the indole ring system, with a dihedral angle of 3.1 (4)°. In contrast, the cyclo­propyl and *p*-tolyl rings are oriented nearly perpendicular to the indole plane, with dihedral angles of 87.0 (3) and 84.49 (9)°, respectively. The cyclo­propyl ring shows the expected angular compression associated with a strained three-membered ring. The torsion angles about the *N*-sulfonyl linkage [S1—N1—C6—C13 = −169.3 (2)° and S1—N1—C15—C14 = 169.2 (2)°] indicate an extended conformation of the *N*-sulfonyl group. In the crystal structure (Fig. 3 ▸), no classical hydrogen bonds are present because the mol­ecule lacks N—H and O—H donor groups. Instead, the crystal packing involves weak C—H⋯O inter­actions, with the sulfonyl oxygen atoms acting as acceptors, together with slipped π–π stacking inter­actions between neighbouring aromatic rings [*Cg*1⋯*Cg*2 = 3.9655 (18) Å, perpendicular distance = 3.627 Å, interplanar angle = 1.10° and slippage = 1.603 Å and *Cg*3⋯*Cg*4 = 3.7798 (18) Å, perpendicular distance = 3.576 Å, interplanar angle = 0.02° and slippage = 1.223 Å where *Cg*1 and *Cg*2 are the centroids of the C7–C10/C18/C19 ring and its syemmetry-generated counterpart at (*x*, 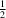 − *y*, −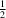 + *z*) and *Cg*2 and *Cg*3 are the centroids of the C3–C6/C12/C13 ring and its symmetry-generated counterpart at (1 − *x*, −*y*, −*z*)].

**Figure 4:**
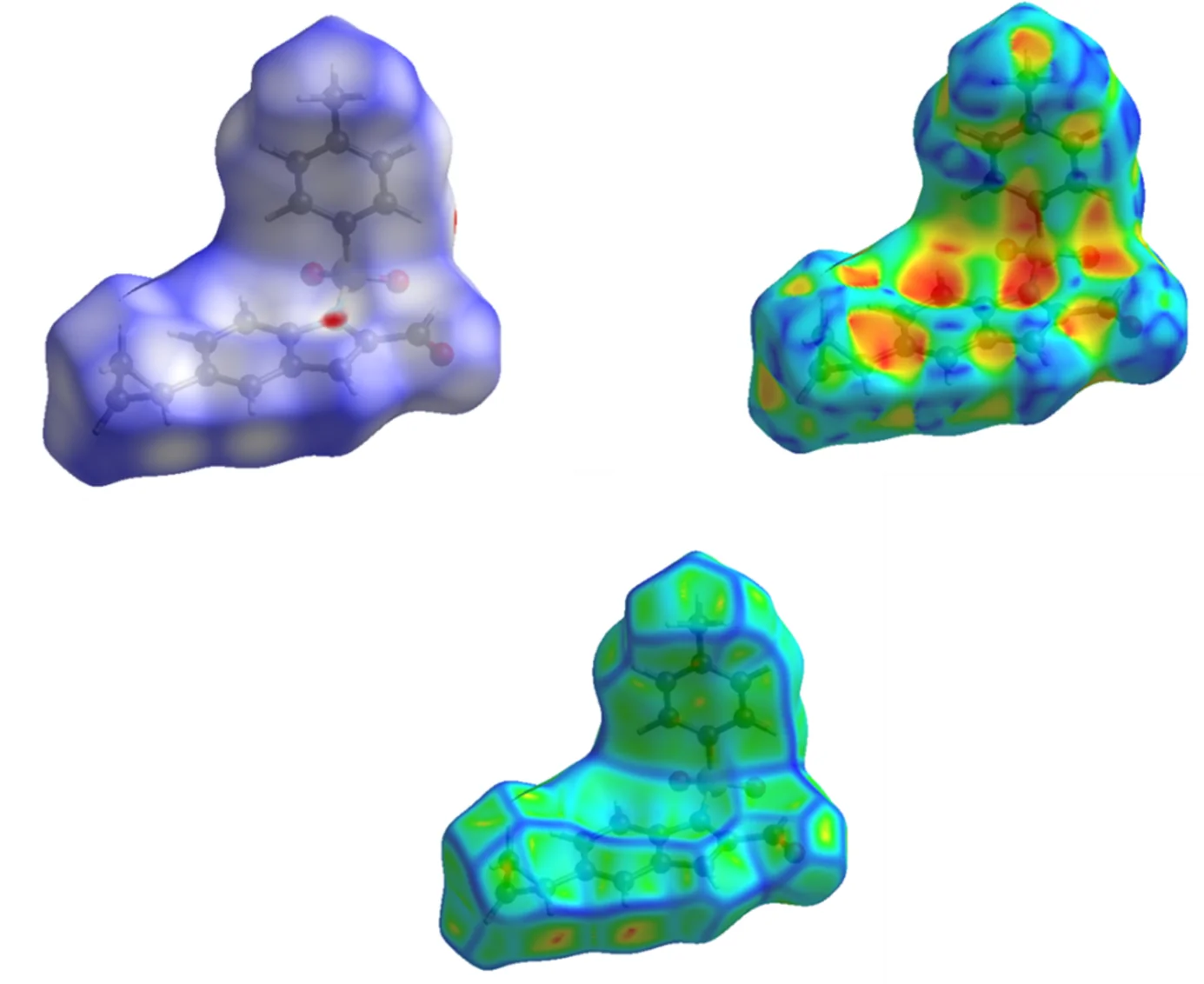
Hirshfeld surface analysis of title compound showing (*a*) *d*_norm_ surface highlighting close inter/intra mol­ecular contacts as red patches, (*b*) shape-index map illustrating complementary red and blue triangular regions associated with slipped aromatic π–π stacking inter­actions and (*c*) curvedness surface showing flat aromatic regions characteristic of stacking inter­actions

The inter­molecular contacts were further analysed using Hirshfeld surface analysis. The *d*_norm_ surface (Fig. 4 ▸) shows prominent red regions corresponding to close C—H⋯O contacts, while the shape-index surface indicates slipped π–π stacking inter­actions. The corresponding two-dimensional fingerprint plots (Fig. 5) show that H⋯H contacts contribute 46.6% to the Hirshfeld surface, followed by O⋯H/H⋯O (23.5%), C⋯H/H⋯C (16.0%) and C⋯C (8.0%). These contacts represent the major contributions to the crystal packing.

A comparison with the related structure of 3-methyl-1-tosyl-1*H*-indole-2-carbaldehyde (Pradeep *et al.*, 2013 ▸) shows that both compounds have a non-coplanar arrangement associated with the *N*-tosyl substituent. However, the title compound adopts a more extended conformation about the *N*-sulfonyl linkage, which may be associated with the presence of the cyclo­propyl substituent at the C5 position. Although *N*-tosyl cyclo­propyl-substituted indole derivatives have been reported in the patent literature (World Intellectual Property Organization, 2011 ▸), closely related crystal structures have not been reported. The present structure therefore adds to the available crystallographic data for cyclo­propyl-substituted *N*-tosyl indole derivatives.

## Synthesis and crystallization

Ethyl 5-*cyclo*­propyl-1-tosyl-1*H*-indole-2-carbox­ylate (5.0 g, 13.03 mmol) was dissolved in 2-meth­yltetra­hydro­furan (50 ml) and cooled to 0°C under a nitro­gen atmosphere. Lithium aluminium hydride (1.2 g, 32.60 mmol, 2.5 equiv.) was added portion wise over 1 h with continuous stirring. The reaction mixture was maintained at 0°C for an additional 1 h until the reduction was complete, as monitored by thin-layer chromatography (TLC). The excess lithium aluminium hydride was carefully quenched with saturated aqueous potassium sodium tartrate solution, followed by the addition of ethyl acetate. The reaction mixture was allowed to warm to room temperature, and the organic layer was separated, washed with saturated potassium sodium tartrate solution and brine, dried over anhydrous sodium sulfate, and concentrated under reduced pressure to afford crude (5-*cyclo*­propyl-1-tosyl-1*H*-indol-2-yl)methanol, which was used directly in the subsequent oxidation step without further purification. The crude alcohol (3.5 g, 10.26 mmol) was dissolved in di­chloro­methane (35 ml) and cooled to 0°C. Dess–Martin periodinane (8.7 g, 20.52 mmol, 2.0 equiv.) was added portionwise over 30 min, and the reaction mixture was stirred at 0°C for 3 h. Upon completion of the reaction (TLC), the precipitated by-product was removed by filtration, and the filtrate was concentrated under reduced pressure. The residue was purified by silica-gel column chromatography to afford the title compound, 5-cyclo­propyl-1-[(4-methyl­benzene)­sulfon­yl]indole-2-carbaldehyde, as a white solid in 92% yield (3.2 g). Crystals suitable for single-crystal X-ray diffraction analysis were obtained by slow evaporation of an ethyl acetate/hexane (9:1 *v*/*v*) solution of the purified compound at room temperature over several days..

## Refinement

Crystal data, data collection, and structure refinement details are given in Table 1 ▸.

## Supplementary Material

Crystal structure: contains datablock(s) I. DOI: 10.1107/S2414314626009272/bx4042sup1.cif

Structure factors: contains datablock(s) I. DOI: 10.1107/S2414314626009272/bx4042Isup2.hkl

Supporting information file. DOI: 10.1107/S2414314626009272/bx4042Isup3.cml

CCDC reference: 2555761

Additional supporting information:  crystallographic information; 3D view; checkCIF report

## Figures and Tables

**Table 1:** Experimental details

Crystal data	
Chemical formula	C_19_H_17_NO_3_S
*M*_r_	339.39
Crystal system, space group	Monoclinic, *P*2_1_/*c*
Temperature (K)	273
*a*, *b*, *c* (Å)	9.9586 (4), 21.0209 (9), 7.9204 (3)
β (°)	99.126 (2)
*V* (Å^3^)	1637.06 (11)
*Z*	4
Radiation type	Mo *K*α
μ (mm^−1^)	0.22
Crystal size (mm)	0.32 × 0.06 × 0.05

Data collection	
Diffractometer	Bruker D8 Quest ECO
Absorption correction	Multi-scan (*SADABS*; Krause *et al.*, 2015 ▸)
*T*_min_, *T*_max_	0.680, 0.746
No. of measured, independent and observed [*I* > 2σ(*I*)] reflections	83410, 4049, 2668
*R*_int_	0.064
(sin θ/λ)_max_ (Å^−1^)	0.668

Refinement	
*R*[*F*^2^ > 2σ(*F*^2^)], *wR*(*F*^2^), *S*	0.065, 0.184, 1.06
No. of reflections	4049
No. of parameters	218
H-atom treatment	H-atom parameters constrained
Δρ_max_, Δρ_min_ (e Å^−3^)	0.41, −0.23

Computer programs: *APEX4* and *SAINT* (Bruker, 2016 ▸), *SHELXT2018/2* (Sheldrick, 2015*a* ▸), *SHELXL* (Sheldrick, 2015*b* ▸) and *OLEX2* (Dolomanov *et al.*, 2009 ▸).

## full crystallographic data

### 5-Cyclopropyl-1-[(4-methylbenzene)sulfonyl]indole-2-carbaldehyde Crystal data

**Table:**

C_19_H_17_NO_3_S	*F*(000) = 712
*M**_r_* = 339.39	*D*_x_ = 1.377 Mg m^−^^3^
Monoclinic, *P*2_1_/*c*	Mo *K*α radiation, λ = 0.71073 Å
*a* = 9.9586 (4) Å	Cell parameters from 9880 reflections
*b* = 21.0209 (9) Å	θ = 2.3–23.3°
*c* = 7.9204 (3) Å	µ = 0.22 mm^−^^1^
β = 99.126 (2)°	*T* = 273 K
*V* = 1637.06 (11) Å^3^	Needle, colourless
*Z* = 4	0.32 × 0.06 × 0.05 mm

### 5-Cyclopropyl-1-[(4-methylbenzene)sulfonyl]indole-2-carbaldehyde Data collection

**Table:**

Bruker D8 Quest ECO diffractometer	2668 reflections with *I* > 2σ(*I*)
ω scans	*R*_int_ = 0.064
Absorption correction: multi-scan (SADABS; Krause *et al.*, 2015)	θ_max_ = 28.3°, θ_min_ = 2.8°
*T*_min_ = 0.680, *T*_max_ = 0.746	*h* = −13→13
83410 measured reflections	*k* = −27→27
4049 independent reflections	*l* = −10→10

### 5-Cyclopropyl-1-[(4-methylbenzene)sulfonyl]indole-2-carbaldehyde Refinement

**Table:**

Refinement on *F*^2^	0 restraints
Least-squares matrix: full	Hydrogen site location: inferred from neighbouring sites
*R*[*F*^2^ > 2σ(*F*^2^)] = 0.065	H-atom parameters constrained
*wR*(*F*^2^) = 0.184	*w* = 1/[σ^2^(*F*_o_^2^) + (0.0656*P*)^2^ + 1.6942*P*] where *P* = (*F*_o_^2^ + 2*F*_c_^2^)/3
*S* = 1.06	(Δ/σ)_max_ < 0.001
4049 reflections	Δρ_max_ = 0.41 e Å^−^^3^
218 parameters	Δρ_min_ = −0.23 e Å^−^^3^

### 5-Cyclopropyl-1-[(4-methylbenzene)sulfonyl]indole-2-carbaldehyde Special details

**Table:**

Geometry. All esds (except the esd in the dihedral angle between two l.s. planes) are estimated using the full covariance matrix. The cell esds are taken into account individually in the estimation of esds in distances, angles and torsion angles; correlations between esds in cell parameters are only used when they are defined by crystal symmetry. An approximate (isotropic) treatment of cell esds is used for estimating esds involving l.s. planes.
Refinement. The structure was solved by direct methods and refined by full-matrix least-squares on (F^2). All non-hydrogen atoms were refined with anisotropic displacement parameters. Hydrogen atoms were placed in calculated positions and refined using a riding model.

### 5-Cyclopropyl-1-[(4-methylbenzene)sulfonyl]indole-2-carbaldehyde Fractional atomic coordinates and isotropic or equivalent isotropic displacement parameters (Å^2^)

**Table:**

	*x*	*y*	*z*	*U*_iso_*/*U*_eq_	
S1	0.34851 (8)	0.64905 (4)	0.12355 (9)	0.0496 (2)	
O1	0.2746 (3)	0.59482 (11)	0.0534 (3)	0.0642 (6)	
O2	0.4513 (2)	0.67646 (12)	0.0409 (3)	0.0647 (6)	
O3	0.7097 (3)	0.73354 (14)	0.4654 (4)	0.0819 (8)	
N1	0.4263 (2)	0.62609 (12)	0.3185 (3)	0.0488 (6)	
C1	0.1366 (5)	0.4586 (2)	0.8626 (6)	0.0876 (13)	
H1A	0.065643	0.479533	0.783852	0.105*	
H1B	0.123379	0.459031	0.981323	0.105*	
C2	0.2763 (4)	0.46231 (19)	0.8264 (5)	0.0709 (10)	
H2	0.345081	0.462744	0.929707	0.085*	
C3	0.3137 (3)	0.50271 (15)	0.6843 (4)	0.0534 (8)	
C4	0.2355 (3)	0.50178 (15)	0.5196 (5)	0.0581 (8)	
H4	0.161396	0.474363	0.499190	0.070*	
C5	0.2635 (3)	0.53947 (15)	0.3878 (4)	0.0554 (8)	
H5	0.210914	0.537331	0.279871	0.066*	
C6	0.3733 (3)	0.58092 (13)	0.4215 (4)	0.0452 (7)	
C7	0.2339 (3)	0.70821 (14)	0.1642 (3)	0.0434 (6)	
C8	0.2692 (3)	0.77164 (15)	0.1546 (4)	0.0527 (8)	
H8	0.353357	0.783046	0.127150	0.063*	
C9	0.1777 (3)	0.81772 (15)	0.1863 (4)	0.0560 (8)	
H9	0.200572	0.860374	0.178169	0.067*	
C10	0.0530 (3)	0.80202 (15)	0.2298 (4)	0.0501 (7)	
C11	−0.0452 (4)	0.85274 (18)	0.2633 (5)	0.0729 (10)	
H11A	0.003942	0.890643	0.302204	0.109*	
H11B	−0.095984	0.838329	0.349431	0.109*	
H11C	−0.106534	0.861729	0.159851	0.109*	
C12	0.4228 (3)	0.54307 (16)	0.7145 (4)	0.0537 (8)	
H12	0.476977	0.543870	0.821508	0.064*	
C13	0.4530 (3)	0.58307 (14)	0.5844 (4)	0.0459 (7)	
C14	0.5534 (3)	0.63084 (16)	0.5795 (4)	0.0516 (7)	
H14	0.620262	0.642139	0.670245	0.062*	
C15	0.5361 (3)	0.65718 (14)	0.4221 (4)	0.0477 (7)	
C16	0.6178 (3)	0.70994 (17)	0.3694 (5)	0.0623 (9)	
H16	0.597420	0.725422	0.258265	0.075*	
C17	0.2027 (4)	0.40283 (19)	0.8008 (6)	0.0802 (12)	
H17A	0.229813	0.368677	0.881351	0.096*	
H17B	0.172129	0.389161	0.684055	0.096*	
C18	0.1090 (3)	0.69125 (15)	0.2069 (4)	0.0538 (8)	
H18	0.085492	0.648608	0.213329	0.065*	
C19	0.0200 (3)	0.73828 (16)	0.2397 (5)	0.0570 (8)	
H19	−0.063568	0.726965	0.268957	0.068*	

### 5-Cyclopropyl-1-[(4-methylbenzene)sulfonyl]indole-2-carbaldehyde Atomic displacement parameters (Å^2^)

**Table:**

	*U*^11^	*U*^22^	*U*^33^	*U*^12^	*U*^13^	*U*^23^
S1	0.0553 (5)	0.0524 (5)	0.0420 (4)	0.0031 (3)	0.0110 (3)	−0.0025 (3)
O1	0.0828 (17)	0.0564 (14)	0.0514 (13)	−0.0019 (12)	0.0040 (12)	−0.0114 (11)
O2	0.0645 (14)	0.0806 (16)	0.0544 (13)	0.0058 (12)	0.0261 (11)	0.0075 (12)
O3	0.0610 (15)	0.0894 (19)	0.095 (2)	−0.0249 (15)	0.0098 (14)	−0.0019 (16)
N1	0.0475 (14)	0.0501 (14)	0.0489 (14)	−0.0005 (11)	0.0076 (11)	0.0024 (11)
C1	0.096 (3)	0.070 (3)	0.110 (3)	−0.005 (2)	0.056 (3)	0.005 (2)
C2	0.073 (2)	0.073 (2)	0.067 (2)	−0.009 (2)	0.0125 (19)	0.0067 (19)
C3	0.0576 (19)	0.0488 (17)	0.0557 (18)	0.0072 (14)	0.0145 (15)	0.0011 (14)
C4	0.0556 (19)	0.0484 (17)	0.072 (2)	−0.0089 (15)	0.0136 (16)	−0.0055 (16)
C5	0.0571 (19)	0.0522 (18)	0.0548 (18)	−0.0033 (15)	0.0028 (15)	−0.0014 (15)
C6	0.0446 (15)	0.0412 (15)	0.0506 (16)	0.0060 (12)	0.0104 (13)	−0.0029 (12)
C7	0.0435 (15)	0.0478 (16)	0.0381 (14)	−0.0008 (13)	0.0038 (11)	0.0004 (12)
C8	0.0483 (17)	0.0512 (17)	0.0603 (19)	−0.0093 (14)	0.0140 (14)	0.0033 (14)
C9	0.0591 (19)	0.0427 (17)	0.066 (2)	−0.0051 (14)	0.0088 (16)	0.0034 (14)
C10	0.0479 (16)	0.0542 (18)	0.0459 (16)	0.0042 (14)	0.0007 (13)	−0.0024 (13)
C11	0.063 (2)	0.069 (2)	0.086 (3)	0.0138 (18)	0.0095 (19)	−0.007 (2)
C12	0.0467 (16)	0.0608 (19)	0.0535 (18)	0.0041 (15)	0.0073 (14)	0.0036 (15)
C13	0.0384 (15)	0.0511 (16)	0.0494 (16)	0.0096 (12)	0.0105 (12)	−0.0016 (13)
C14	0.0362 (14)	0.0627 (19)	0.0552 (18)	0.0026 (14)	0.0049 (13)	−0.0035 (15)
C15	0.0376 (15)	0.0506 (17)	0.0557 (17)	0.0064 (13)	0.0100 (13)	−0.0019 (14)
C16	0.0531 (19)	0.065 (2)	0.070 (2)	−0.0043 (17)	0.0148 (17)	0.0002 (18)
C17	0.097 (3)	0.058 (2)	0.093 (3)	−0.006 (2)	0.039 (2)	0.003 (2)
C18	0.0489 (17)	0.0434 (16)	0.070 (2)	−0.0089 (13)	0.0128 (15)	0.0018 (15)
C19	0.0381 (15)	0.060 (2)	0.073 (2)	−0.0063 (14)	0.0110 (15)	0.0036 (16)

### 5-Cyclopropyl-1-[(4-methylbenzene)sulfonyl]indole-2-carbaldehyde Geometric parameters (Å, º)

**Table:**

S1—O1	1.421 (2)	C7—C18	1.386 (4)
S1—O2	1.422 (2)	C8—H8	0.9300
S1—N1	1.684 (3)	C8—C9	1.380 (4)
S1—C7	1.752 (3)	C9—H9	0.9300
O3—C16	1.201 (4)	C9—C10	1.380 (4)
N1—C6	1.409 (4)	C10—C11	1.499 (5)
N1—C15	1.418 (4)	C10—C19	1.385 (4)
C1—H1A	0.9700	C11—H11A	0.9600
C1—H1B	0.9700	C11—H11B	0.9600
C1—C2	1.467 (5)	C11—H11C	0.9600
C1—C17	1.467 (6)	C12—H12	0.9300
C2—H2	0.9800	C12—C13	1.399 (4)
C2—C3	1.503 (5)	C13—C14	1.422 (4)
C2—C17	1.447 (5)	C14—H14	0.9300
C3—C4	1.409 (5)	C14—C15	1.350 (4)
C3—C12	1.370 (5)	C15—C16	1.474 (5)
C4—H4	0.9300	C16—H16	0.9300
C4—C5	1.374 (5)	C17—H17A	0.9700
C5—H5	0.9300	C17—H17B	0.9700
C5—C6	1.390 (4)	C18—H18	0.9300
C6—C13	1.404 (4)	C18—C19	1.380 (4)
C7—C8	1.384 (4)	C19—H19	0.9300
			
O1—S1—O2	120.73 (15)	C8—C9—H9	119.2
O1—S1—N1	105.26 (14)	C8—C9—C10	121.6 (3)
O1—S1—C7	109.21 (14)	C10—C9—H9	119.2
O2—S1—N1	106.15 (14)	C9—C10—C11	120.8 (3)
O2—S1—C7	109.53 (15)	C9—C10—C19	118.4 (3)
N1—S1—C7	104.68 (13)	C19—C10—C11	120.8 (3)
C6—N1—S1	124.3 (2)	C10—C11—H11A	109.5
C6—N1—C15	107.5 (2)	C10—C11—H11B	109.5
C15—N1—S1	126.8 (2)	C10—C11—H11C	109.5
H1A—C1—H1B	115.0	H11A—C11—H11B	109.5
C2—C1—H1A	117.9	H11A—C11—H11C	109.5
C2—C1—H1B	117.9	H11B—C11—H11C	109.5
C2—C1—C17	59.1 (3)	C3—C12—H12	120.0
C17—C1—H1A	117.9	C3—C12—C13	120.1 (3)
C17—C1—H1B	117.9	C13—C12—H12	120.0
C1—C2—H2	113.4	C6—C13—C14	107.2 (3)
C1—C2—C3	122.1 (4)	C12—C13—C6	120.1 (3)
C3—C2—H2	113.4	C12—C13—C14	132.8 (3)
C17—C2—C1	60.4 (3)	C13—C14—H14	125.4
C17—C2—H2	113.4	C15—C14—C13	109.1 (3)
C17—C2—C3	124.3 (4)	C15—C14—H14	125.4
C4—C3—C2	121.3 (3)	N1—C15—C16	126.3 (3)
C12—C3—C2	120.2 (3)	C14—C15—N1	108.6 (3)
C12—C3—C4	118.4 (3)	C14—C15—C16	125.1 (3)
C3—C4—H4	118.4	O3—C16—C15	122.1 (4)
C5—C4—C3	123.1 (3)	O3—C16—H16	119.0
C5—C4—H4	118.4	C15—C16—H16	119.0
C4—C5—H5	121.2	C1—C17—H17A	117.7
C4—C5—C6	117.6 (3)	C1—C17—H17B	117.7
C6—C5—H5	121.2	C2—C17—C1	60.4 (3)
C5—C6—N1	131.8 (3)	C2—C17—H17A	117.7
C5—C6—C13	120.6 (3)	C2—C17—H17B	117.7
C13—C6—N1	107.5 (3)	H17A—C17—H17B	114.8
C8—C7—S1	119.7 (2)	C7—C18—H18	120.3
C8—C7—C18	120.4 (3)	C19—C18—C7	119.3 (3)
C18—C7—S1	119.9 (2)	C19—C18—H18	120.3
C7—C8—H8	120.5	C10—C19—H19	119.4
C9—C8—C7	119.0 (3)	C18—C19—C10	121.2 (3)
C9—C8—H8	120.5	C18—C19—H19	119.4
			
S1—N1—C6—C5	9.9 (5)	C4—C5—C6—N1	−178.3 (3)
S1—N1—C6—C13	−169.3 (2)	C4—C5—C6—C13	0.9 (4)
S1—N1—C15—C14	169.2 (2)	C5—C6—C13—C12	0.3 (4)
S1—N1—C15—C16	−10.2 (4)	C5—C6—C13—C14	−178.3 (3)
S1—C7—C8—C9	179.8 (2)	C6—N1—C15—C14	2.2 (3)
S1—C7—C18—C19	179.6 (3)	C6—N1—C15—C16	−177.1 (3)
O1—S1—N1—C6	−31.3 (3)	C6—C13—C14—C15	0.4 (3)
O1—S1—N1—C15	163.8 (2)	C7—S1—N1—C6	83.8 (3)
O1—S1—C7—C8	−149.7 (2)	C7—S1—N1—C15	−81.1 (3)
O1—S1—C7—C18	30.7 (3)	C7—C8—C9—C10	1.0 (5)
O2—S1—N1—C6	−160.3 (2)	C7—C18—C19—C10	0.4 (5)
O2—S1—N1—C15	34.8 (3)	C8—C7—C18—C19	0.0 (5)
O2—S1—C7—C8	−15.4 (3)	C8—C9—C10—C11	−179.8 (3)
O2—S1—C7—C18	165.0 (2)	C8—C9—C10—C19	−0.6 (5)
N1—S1—C7—C8	98.0 (3)	C9—C10—C19—C18	−0.1 (5)
N1—S1—C7—C18	−81.6 (3)	C11—C10—C19—C18	179.1 (3)
N1—C6—C13—C12	179.7 (3)	C12—C3—C4—C5	0.0 (5)
N1—C6—C13—C14	1.0 (3)	C12—C13—C14—C15	−178.1 (3)
N1—C15—C16—O3	−179.8 (3)	C13—C14—C15—N1	−1.6 (3)
C1—C2—C3—C4	−46.6 (5)	C13—C14—C15—C16	177.8 (3)
C1—C2—C3—C12	131.3 (4)	C14—C15—C16—O3	1.0 (5)
C2—C3—C4—C5	177.8 (3)	C15—N1—C6—C5	177.3 (3)
C2—C3—C12—C13	−176.6 (3)	C15—N1—C6—C13	−2.0 (3)
C3—C2—C17—C1	−110.6 (5)	C17—C1—C2—C3	114.1 (4)
C3—C4—C5—C6	−1.0 (5)	C17—C2—C3—C4	27.3 (5)
C3—C12—C13—C6	−1.4 (4)	C17—C2—C3—C12	−154.9 (4)
C3—C12—C13—C14	176.8 (3)	C18—C7—C8—C9	−0.7 (5)
C4—C3—C12—C13	1.3 (5)		
